# Intraoperative Mitigation Strategies Aimed at Reducing Clinically Relevant Postoperative Pancreatic Fistula After Pancreaticoduodenectomy: A Single-Center Retrospective Cohort Study

**DOI:** 10.7759/cureus.110876

**Published:** 2026-06-15

**Authors:** Luca Ottaviani, Marta Morelli, Andrea Celotti, Roberto Grassia, Gian Luca Baiocchi

**Affiliations:** 1 General Surgery, ASST Cremona, Cremona, ITA; 2 Clinical and Experimental Sciences, University of Brescia, Brescia, ITA; 3 Digestive Endoscopy, ASST Cremona, Cremona, ITA

**Keywords:** fistula risk score, negative-pressure drainage, pancreatic anastomosis, pancreatic fistula, pancreaticoduodenectomy, pancreatic stent, percutaneous transhepatic biliary drainage, postoperative pancreatic fistula

## Abstract

Background

Clinically relevant postoperative pancreatic fistula (CR-POPF) remains one of the most important drivers of morbidity after pancreaticoduodenectomy (PD). Several intraoperative adjuncts have been proposed to protect the pancreatic anastomosis or reduce ductal and biliopancreatic pressure, but their comparative real-world impact remains uncertain.

Methods

We conducted a retrospective single-center cohort study of consecutive adult patients who underwent PD at ASST Cremona between December 2021 and January 2026. Patients were stratified according to the 2016 International Study Group of Pancreatic Surgery classification into no POPF, Grade B POPF, and Grade C POPF. The primary endpoint was CR-POPF, defined as Grade B or C POPF. Intraoperative adjuncts of interest included intraoperative percutaneous transhepatic biliary drainage (PTBD), sponge-assisted negative-pressure drainage, coronary stent support of the pancreatic anastomosis, and externalized Wirsung duct stenting. Secondary outcomes included major morbidity (Clavien-Dindo ≥3a), postoperative interventions, reoperation, unplanned ICU admission, length of stay, readmission, and 30-day mortality.

Results

Seventy-six patients were included. Overall, 52 patients (68.4%) did not develop POPF, while 24 (31.6%) developed CR-POPF, including 18 Grade B fistulas (23.7%) and six Grade C fistulas (7.9%). A soft pancreatic remnant, a smaller main pancreatic duct diameter, and a higher Fistula Risk Score (FRS) were significantly associated with POPF. Intraoperative PTBD was used in 56 patients (73.7%), externalized Wirsung duct stenting in 40 (52.6%), coronary stenting in 35 (46.1%), and sponge-assisted drainage in five (6.6%). In exploratory parsimonious logistic regression models, none of the adjunctive strategies was significantly associated with reduced CR-POPF. Low FRS (1-2) was protective against CR-POPF (OR: 0.19; 95% CI: 0.05-0.66; p = 0.009). CR-POPF was associated with longer postoperative stay, higher major morbidity, more abdominal collections, greater need for interventional radiology or endoscopic treatment, reoperation, unplanned ICU admission, and higher 30-day mortality in Grade C cases.

Conclusions

In this single-center cohort, CR-POPF after PD was mainly associated with pancreatic texture, duct size, and FRS rather than with any single intraoperative adjunct. These findings support individualized, risk-stratified anastomotic protection and highlight the need for larger prospective studies to clarify which patients may benefit from specific technical adjuncts.

## Introduction

Pancreaticoduodenectomy (PD) remains the standard potentially curative surgical procedure for malignancies of the pancreatic head, distal bile duct, ampulla, and periampullary region, as well as for selected benign and premalignant diseases [1]. Despite advances in operative technique and perioperative care, PD continues to be associated with substantial postoperative morbidity. Among procedure-specific complications, postoperative pancreatic fistula (POPF) is widely regarded as one of the most important determinants of adverse postoperative outcomes, including prolonged hospitalization, reintervention, organ failure, readmission, and mortality [2]. The 2016 update of the International Study Group of Pancreatic Surgery definition reclassified the former Grade A fistula as a biochemical leak and reserved the term “clinically relevant POPF” (CR-POPF) for Grade B and Grade C events [3]. This distinction is clinically meaningful because CR-POPF may lead to prolonged drainage, intra-abdominal collections, hemorrhage, sepsis, reintervention, delayed discharge, readmission, and mortality. The Clavien-Dindo classification provides a complementary framework for grading the severity of postoperative morbidity and the invasiveness of required treatments [4].

Prediction of POPF risk is central to perioperative decision-making. The Fistula Risk Score (FRS), based on pancreatic texture, duct diameter, pathology, and blood loss, remains one of the most commonly used tools for intraoperative risk stratification after PD [5,6]. Soft gland texture and small duct diameter are consistently associated with an increased risk of CR-POPF, emphasizing that anastomotic failure is influenced by both surgical technique and underlying pancreatic biology.

Several intraoperative strategies have been developed to mitigate POPF risk. These include internal or external pancreatic duct stents, specific pancreaticojejunostomy configurations, anastomotic reinforcement devices, biliary decompression strategies, and negative-pressure drainage systems. However, evidence remains heterogeneous, and no single method has achieved universal acceptance across surgical centers [7-12]. In real-world practice, the selected technique is frequently individualized according to gland texture, duct size, anastomotic configuration, surgeon preference, and institutional protocols.

The present study evaluated a single-center experience with intraoperative adjunctive strategies used during PD. Specifically, we assessed whether intraoperative percutaneous transhepatic biliary drainage (PTBD), sponge-assisted negative-pressure drainage, coronary stent support of the pancreatic anastomosis, or externalized Wirsung duct stenting was associated with the incidence and severity of CR-POPF. Given the retrospective design, limited sample size, and non-randomized use of these techniques, the study was not intended as a confirmatory comparative effectiveness analysis. Rather, the primary objective was to explore real-world associations between adjunctive intraoperative strategies and CR-POPF, while secondary exploratory analyses assessed pancreatic risk factors, FRS categories, and reconstruction techniques as potential factors associated with CR-POPF.

## Materials and methods

Study design and setting

The use of adjunctive intraoperative strategies was not protocol-mandated but was based on the operating surgeon’s judgment. The decision was guided by intraoperative risk factors, including pancreatic texture, main pancreatic duct diameter, local anatomy, technical difficulty of the pancreaticojejunostomy, quality of the pancreatic remnant, and perceived risk of anastomotic failure. Therefore, these adjuncts were used as individualized risk-mitigation measures rather than as standardized interventions applied according to a predefined algorithm. The adjunctive strategies of interest were intraoperative PTBD, sponge-assisted negative-pressure drainage, coronary stent support of the pancreatic anastomosis, and externalized Wirsung duct stenting.

Externalized Wirsung duct stenting was generally considered in patients with a soft pancreatic remnant, small duct, or technically demanding pancreaticojejunostomy. After identification and calibration of the main pancreatic duct, a 5-7.5 Fr catheter was inserted transanastomotically, advanced through the jejunal limb, exteriorized through the abdominal wall, and connected to a collection bag, with the aim of decompressing the pancreatic duct and diverting pancreatic juice away from the anastomosis. Removal was planned according to the postoperative course, usually after several weeks.

Coronary stent support was used as an internal transanastomotic device in selected high-risk anastomoses. After duct calibration, a balloon-expandable coronary-type stent was positioned across the pancreatic duct and pancreaticojejunostomy and then deployed to provide internal radial support across the anastomosis. No external removal was required. Intraoperative PTBD was defined as the maintenance or placement of a transhepatic biliary drainage catheter during PD when biliary decompression was considered useful, particularly after preoperative drainage or in patients with jaundice, cholangitis, delayed surgery, or high-risk biliary reconstruction. Sponge-assisted negative-pressure drainage was reserved for highly selected cases and consisted of positioning a sponge connected to a drain near the pancreatic anastomosis, with postoperative negative-pressure suction to limit local fluid accumulation and support controlled drainage.

Data collection

Data were collected anonymously in a dedicated database. Preoperative variables included age, sex, BMI, American Society of Anesthesiologists (ASA) score, age-adjusted Charlson Comorbidity Index, diabetes mellitus, chronic obstructive pulmonary disease, ischemic heart disease, chronic renal failure, and neoadjuvant therapy. Pathological variables included final histological diagnosis and pathological TNM stage when applicable.

Intraoperative variables included operative approach, operative time, estimated blood loss, transfusion requirement, vascular resection, multiorgan resection, pancreatic texture, main pancreatic duct diameter, conversion, pancreatic reconstruction technique, digestive reconstruction, jejunostomy placement, drain placement, and the use of the adjunctive strategies of interest.

Postoperative variables included drain amylase values on postoperative days 1, 3, and 5; drain amylase greater than three times the upper limit of normal on postoperative day 3; hospital length of stay; postoperative transfusions; Clavien-Dindo complication grade; FRS; POPF grade; drain at discharge; wound infection; lymphatic fistula; nasogastric tube requirement; delayed gastric emptying; biliary fistula; postpancreatectomy hemorrhage; abdominal collections; need for interventional radiology, endoscopic treatment, or reoperation; unplanned ICU admission; sepsis; readmission; and 30-day mortality. The FRS was calculated when all required components were available.

Statistical analysis

Continuous variables were reported as mean and SD for descriptive consistency. Given the limited sample size and the expected non-normal distribution of several perioperative variables, comparisons across the no POPF, Grade B POPF, and Grade C POPF groups were performed using the Kruskal-Wallis test, with the H statistic and corresponding p-value reported. Categorical variables were compared using the chi-square test or Fisher’s exact test, as appropriate according to expected cell counts. For chi-square tests, χ² statistics, degrees of freedom, and p-values were reported. For Fisher’s exact tests, exact p-values were reported, and the test used was specified in the table legend.

Logistic regression was used to explore factors associated with CR-POPF. Given the limited number of CR-POPF events, multivariable models were intentionally parsimonious and should be interpreted as exploratory. Results were reported as ORs with 95% CIs, p-values, and Wald statistics. No correction for multiple testing was performed. Statistical significance was set at p < 0.05. Analyses were performed using Microsoft Excel 365 (Microsoft Corporation, Redmond, WA, USA) and jamovi (version 2.6; The jamovi Project, Sydney, Australia).

## Results

Intraoperative findings and risk stratification

A total of 76 patients underwent PD during the study period. Among them, 52 (68.4%) had no POPF, 18 (23.7%) developed Grade B POPF, and six (7.9%) developed Grade C POPF. The mean age was 69.9 ± 10.4 years, 36 patients (47.4%) were male, and the mean BMI was 24.2 ± 4.5 kg/m². Most patients had an ASA score ≥3 (71.1%). Pancreatic ductal adenocarcinoma was the most frequent diagnosis (48/76, 63.2%). Baseline demographic and clinical variables did not differ significantly across POPF groups (Table 1).

**Table 1:** Baseline demographic and clinical characteristics stratified by POPF grade Continuous variables are reported as mean ± SD and were compared across POPF groups using the Kruskal-Wallis test. Categorical variables were compared using the Pearson chi-square test. ASA, American Society of Anesthesiologists; POPF, postoperative pancreatic fistula

Variable	Overall (n = 76)	No POPF (n = 52)	POPF B (n = 18)	POPF C (n = 6)	Test statistic	p-value
Age, years, mean ± SD	69.9 ± 10.4	70.1 ± 9.9	67.3 ± 12.1	75.7 ± 7.9	H (2) = 2.94	0.230
BMI, kg/m², mean ± SD	24.2 ± 4.5	23.7 ± 4.5	25.5 ± 4.9	24.2 ± 1.5	H (2) = 2.13	0.344
Male sex	36 (47.4%)	24 (46.2%)	10 (55.6%)	2 (33.3%)	χ² (2) = 0.99	0.610
ASA score ≥3	54 (71.1%)	34 (65.4%)	14 (77.8%)	6 (100.0%)	χ² (2) = 3.65	0.161
Charlson index ≥5	54 (71.1%)	37 (71.2%)	13 (72.2%)	4 (66.7%)	χ² (2) = 0.07	0.966
Diabetes mellitus	17 (22.4%)	12 (23.1%)	4 (22.2%)	1 (16.7%)	χ² (2) = 0.13	0.938
Neoadjuvant chemotherapy	26 (34.2%)	18 (34.6%)	5 (27.8%)	3 (50.0%)	χ² (2) = 1.00	0.607
Neoadjuvant chemoradiotherapy	5 (6.6%)	4 (7.7%)	1 (5.6%)	0 (0.0%)	χ² (2) = 0.56	0.757
Pancreatic ductal adenocarcinoma	48 (63.2%)	37 (71.2%)	8 (44.4%)	3 (50.0%)	χ² (2) = 4.58	0.101

The operation was performed via an open approach in 66 patients (86.8%) and laparoscopically in 10 (13.2%). Operative time differed significantly across groups (p = 0.022), while estimated blood loss showed borderline significance (p = 0.050). Vascular resection was performed in 19 patients (25.0%) and was less frequent among patients who developed POPF (p = 0.017).

Pancreatic remnant characteristics were strongly associated with POPF. A soft gland was present in 37 patients overall (48.7%) but was observed in 18/18 patients with Grade B POPF and 5/6 patients with Grade C POPF (p < 0.001). The mean main pancreatic duct diameter was smaller in the POPF groups (4.8 ± 1.6 mm in no POPF, 3.6 ± 1.6 mm in Grade B POPF, and 3.8 ± 1.6 mm in Grade C POPF; p = 0.015). Medium-to-high FRS (3-7) was more frequent in patients who developed POPF (p < 0.001). Complete FRS data were available for 59 of 76 patients (77.6%); therefore, FRS-based comparisons and regression models including FRS were performed in the complete-case population.

Mitigation strategies and pancreatic reconstruction

Intraoperative PTBD was the most common adjunctive strategy (56/76, 73.7%), followed by externalized Wirsung duct stenting (40/76, 52.6%), coronary stent support of the pancreatic anastomosis (35/76, 46.1%), and sponge-assisted drainage (5/76, 6.6%). The distribution of these strategies did not differ significantly across POPF groups.

Blumgart pancreaticojejunostomy was the most frequently used reconstruction technique (52/76, 68.4%), followed by invagination pancreaticojejunostomy (14/76, 18.4%), duct-to-mucosa pancreaticojejunostomy (8/76, 10.5%), and pancreatogastrostomy (2/76, 2.6%). Duct-to-mucosa reconstruction showed a non-significant trend across POPF categories (p = 0.064) (Table 2).

**Table 2:** Pancreatic characteristics, FRS categories, mitigation strategies, and reconstruction techniques Continuous variables are reported as mean ± SD and were compared using the Kruskal-Wallis test. Categorical variables were compared using the Pearson chi-square test. FRS categories were reported for patients with complete FRS data. FRS, Fistula Risk Score; PJ, pancreaticojejunostomy; POPF, postoperative pancreatic fistula; PTBD, percutaneous transhepatic biliary drainage

Variable	Overall (n = 76)	No POPF (n = 52)	POPF B (n = 18)	POPF C (n = 6)	Test statistic	p-value
Soft pancreatic remnant	37 (48.7%)	14 (26.9%)	18 (100.0%)	5 (83.3%)	χ² (2) = 31.71	<0.001
Main duct diameter, mm, mean ± SD	4.5 ± 1.7	4.8 ± 1.6	3.6 ± 1.6	3.8 ± 1.6	H (2) = 8.40	0.015
Low FRS (1-2)	22 (28.9%)	20 (38.5%)	1 (5.6%)	1 (16.7%)	χ² (2) = 7.52	0.023
Medium-high FRS (3-7)	37 (48.7%)	16 (30.8%)	16 (88.9%)	5 (83.3%)	χ² (2) = 21.21	<0.001
Intraoperative PTBD	56 (73.7%)	41 (78.8%)	11 (61.1%)	4 (66.7%)	χ² (2) = 2.33	0.311
Sponge-assisted drainage	5 (6.6%)	3 (5.8%)	2 (11.1%)	0 (0.0%)	χ² (2) = 1.08	0.583
Coronary stent	35 (46.1%)	25 (48.1%)	8 (44.4%)	2 (33.3%)	χ² (2) = 0.50	0.781
Externalized Wirsung stent	40 (52.6%)	28 (53.8%)	10 (55.6%)	2 (33.3%)	χ² (2) = 0.99	0.610
Blumgart reconstruction	52 (68.4%)	37 (71.2%)	11 (61.1%)	4 (66.7%)	χ² (2) = 0.63	0.729
Invagination PJ	14 (18.4%)	8 (15.4%)	6 (33.3%)	0 (0.0%)	χ² (2) = 4.34	0.114
Duct-to-mucosa PJ	8 (10.5%)	6 (11.5%)	0 (0.0%)	2 (33.3%)	χ² (2) = 5.49	0.064
Pancreatogastrostomy	2 (2.6%)	1 (1.9%)	1 (5.6%)	0 (0.0%)	χ² (2) = 0.87	0.649

Postoperative outcomes

The development of POPF was associated with substantially worse postoperative outcomes (Table 3). The mean postoperative length of stay was 26.0 ± 14.9 days in patients without POPF, 43.1 ± 28.9 days in those with Grade B POPF, and 52.5 ± 48.3 days in those with Grade C POPF (p = 0.003). Overall postoperative complications were more frequent in the POPF groups (p = 0.019), and major morbidity (Clavien-Dindo ≥3a) increased from 42.3% in patients without POPF to 83.3% in Grade B and 100.0% in Grade C POPF (p = 0.001).

**Table 3:** Postoperative outcomes stratified by POPF grade Continuous variables are reported as mean ± SD and were compared using the Kruskal-Wallis test. Categorical outcomes were compared using the Pearson chi-square test. POPF, postoperative pancreatic fistula

Outcome	Overall (n = 76)	No POPF (n = 52)	POPF B (n = 18)	POPF C (n = 6)	Test statistic	p-value
Postoperative stay, days, mean ± SD	32.1 ± 24.2	26.0 ± 14.9	43.1 ± 28.9	52.5 ± 48.3	H (2) = 11.62	0.003
Any postoperative complication	62 (81.6%)	38 (73.1%)	18 (100.0%)	6 (100.0%)	χ² (2) = 7.92	0.019
Clavien-Dindo ≥3a	43 (56.6%)	22 (42.3%)	15 (83.3%)	6 (100.0%)	χ² (2) = 14.16	0.001
Wound infection	20 (26.3%)	12 (23.1%)	7 (38.9%)	1 (16.7%)	χ² (2) = 2.04	0.361
Drain at discharge	14 (18.4%)	4 (7.7%)	7 (38.9%)	3 (50.0%)	χ² (2) = 12.98	0.002
Abdominal collection	49 (64.5%)	27 (51.9%)	16 (88.9%)	6 (100.0%)	χ² (2) = 11.57	0.003
Interventional radiology	33 (43.4%)	14 (26.9%)	14 (77.8%)	5 (83.3%)	χ² (2) = 18.30	<0.001
Endoscopic treatment	16 (21.1%)	6 (11.5%)	6 (33.3%)	4 (66.7%)	χ² (2) = 11.98	0.003
Surgical reintervention	15 (19.7%)	8 (15.4%)	1 (5.6%)	6 (100.0%)	χ² (2) = 27.31	<0.001
Unplanned ICU admission	20 (26.3%)	9 (17.3%)	5 (27.8%)	6 (100.0%)	χ² (2) = 19.00	<0.001
Readmission	11 (14.5%)	7 (13.5%)	4 (22.2%)	0 (0.0%)	χ² (2) = 1.93	0.381
30-day mortality	5 (6.6%)	2 (3.8%)	0 (0.0%)	3 (50.0%)	χ² (2) = 20.31	<0.001

Abdominal collections, interventional radiology procedures, endoscopic treatment, reoperation, and unplanned ICU admission were all significantly more frequent in patients with POPF. Thirty-day mortality occurred in five patients (6.6%) overall and reached 50.0% in patients with Grade C POPF (p < 0.001).

Exploratory logistic regression models were used to evaluate the association between intraoperative adjunctive strategies, reconstruction techniques, and CR-POPF. In the model including adjunctive strategies and FRS category, low FRS (1-2) was protective against CR-POPF (OR: 0.19; 95% CI: 0.05-0.66; p = 0.009). Intraoperative PTBD, sponge-assisted drainage, coronary stenting, and externalized Wirsung duct stenting were not significantly associated with CR-POPF (Table 4). Similar findings were observed when reconstruction techniques were modeled, with low FRS remaining protective (OR: 0.15; 95% CI: 0.03-0.72; p = 0.018), while Blumgart, invagination, and duct-to-mucosa reconstruction were not significant predictors (Table 5).

**Table 4:** Exploratory logistic regression model including intraoperative adjunctive strategies for CR-POPF CR-POPF, clinically relevant postoperative pancreatic fistula; FRS, Fistula Risk Score; PTBD, percutaneous transhepatic biliary drainage

Predictor	Outcome	OR	95% CI	p-value
PTBD	CR-POPF	0.62	0.16-2.33	0.481
Sponge-assisted drainage	CR-POPF	2.84	0.32-25.24	0.349
Coronary stent	CR-POPF	0.80	0.18-3.55	0.773
Externalized Wirsung stent	CR-POPF	0.73	0.19-2.78	0.646
Low FRS (1-2)	CR-POPF	0.19	0.05-0.66	0.009

**Table 5:** Exploratory logistic regression model including pancreatic reconstruction techniques for CR-POPF CR-POPF, clinically relevant postoperative pancreatic fistula; FRS, Fistula Risk Score

Model/predictor	Outcome	OR	95% CI	p-value
Blumgart	CR-POPF	0.59	0.03-10.23	0.719
Invagination	CR-POPF	1.04	0.05-20.65	0.982
Duct-to-mucosa	CR-POPF	0.69	0.03-18.33	0.822
Low FRS (1-2)	CR-POPF	0.15	0.03-0.72	0.018

## Discussion

This study evaluated the real-world use of intraoperative adjunctive strategies for CR-POPF after PD in a single-center cohort. The principal finding is that none of the analyzed technical adjuncts, including intraoperative PTBD, sponge-assisted drainage, coronary stent support, or externalized Wirsung duct stenting, was associated with a statistically significant reduction in CR-POPF in exploratory risk-adjusted models. In contrast, pancreatic remnant characteristics and FRS categories were strongly associated with fistula development. These results reinforce the concept that POPF risk is multifactorial and cannot be fully explained by the use or non-use of a single technical device.

The observed overall CR-POPF rate was 31.6%, with Grade C events in 7.9% of patients. This rate is higher than that reported in some large multicenter series, but direct comparisons must consider differences in case mix, high-risk pancreatic features, institutional referral patterns, definitions, and completeness of complication capture [13,14]. In the present cohort, nearly all patients with Grade B or Grade C POPF had soft pancreatic texture, and the mean duct diameter was smaller in the POPF groups. These findings are consistent with the established pathophysiology of pancreatic anastomotic failure and with previous validation studies of the FRS [5,15,16].

Duct stenting remains one of the most debated mitigation strategies. Externalized stents may theoretically decompress the main pancreatic duct and divert pancreatic juice away from the anastomosis, while internal stents may facilitate duct identification and maintain local patency. However, published evidence is mixed, with some studies suggesting benefit in selected high-risk patients and others failing to show a consistent reduction in CR-POPF [17-20]. In our cohort, externalized Wirsung duct stenting did not significantly reduce CR-POPF. This absence of statistical significance should not be interpreted as definitive inefficacy, as stents may have been preferentially used in technically challenging or high-risk anastomoses, creating confounding by indication.

The use of coronary stents to support high-risk pancreaticojejunostomy has been described as an innovative technique for securing the anastomosis in selected patients [21,22]. In this cohort, coronary stent placement was not associated with a significant reduction in CR-POPF. The wide CIs observed in regression models suggest that larger datasets are needed to determine whether a specific subgroup could benefit from this approach.

PTBD and other biliary decompression strategies may theoretically reduce pressure in the biliary and pancreaticobiliary system and may facilitate management in selected high-risk contexts. Nevertheless, PTBD in the setting of POPF prevention is not a standardized approach, and available evidence is still emerging [23]. In this analysis, intraoperative PTBD was common but was not significantly associated with lower CR-POPF. Selection bias is plausible because such strategies may have been used more frequently when the surgeon perceived a higher baseline risk.

Sponge-assisted negative-pressure drainage was used in only a small minority of cases. Endoscopic vacuum therapy and negative-pressure approaches have shown utility for gastrointestinal leaks and selected pancreatic fistula management scenarios [24,25]. However, the role of prophylactic or intraoperative sponge-based strategies around pancreatic anastomoses remains insufficiently defined. The very small number of sponge-treated patients in this cohort prevents meaningful conclusions regarding efficacy.

The pancreatic reconstruction technique also did not significantly predict CR-POPF. Blumgart reconstruction was the dominant approach, reflecting contemporary efforts to reduce tension and shear forces at the pancreaticojejunostomy. Prior literature suggests that technique may matter, but comparisons between Blumgart, duct-to-mucosa, invagination pancreaticojejunostomy, and pancreatogastrostomy remain affected by gland texture, duct size, surgeon experience, and patient selection [10-12,26]. Our data support the pragmatic interpretation that reconstruction should be tailored to intraoperative anatomy rather than selected according to a universal hierarchy.

The clinical impact of CR-POPF in this cohort was substantial. Patients with POPF experienced longer postoperative hospitalization, higher major morbidity, more abdominal collections, greater need for interventional radiology and endoscopic treatment, higher reoperation rates, and increased unplanned ICU admission. Grade C POPF was associated with particularly severe outcomes, including 30-day mortality of 50.0%. These findings are consistent with reports identifying CR-POPF, especially Grade C POPF, as a major driver of morbidity and mortality after PD [14,27].

Limitations

This study has several limitations. First, the sample size was limited, particularly for sponge-assisted drainage and subgroup analyses. Second, the retrospective design exposes the analysis to documentation bias and unmeasured confounding. Third, the study was conducted at a single center, and results may reflect local surgical expertise, perioperative pathways, and selection patterns. Fourth, adjunctive strategies and reconstruction techniques were selected according to surgeon judgment rather than a predefined protocol; exact replication of the intraoperative decision-making process is limited, and confounding by indication cannot be excluded. Fifth, wide CIs in several models limit the precision of effect estimates. Sixth, no correction for multiple testing was performed. The regression analyses should therefore be interpreted as exploratory and hypothesis-generating. Despite these limitations, the study reflects real-world decision-making in pancreatic surgery and provides useful data for designing future prospective studies.

Future perspectives

Future research should prioritize prospective multicenter cohorts and randomized or protocolized studies focused on well-defined high-risk subgroups. Integration of FRS, pancreatic texture, duct diameter, radiologic features, drain biomarkers, and intraoperative findings may allow more precise selection of mitigation strategies. The future of POPF prevention is unlikely to depend on a single universal technique; rather, it will probably rely on risk-adapted combinations of anastomotic technique, ductal drainage, local reinforcement, drainage management, and early postoperative detection algorithms.

## Conclusions

In this single-center retrospective cohort of patients undergoing PD, CR-POPF occurred in 31.6% of cases and was associated with major adverse postoperative outcomes. Soft pancreatic texture, smaller main pancreatic duct diameter, and higher FRS were strongly associated with POPF, whereas the analyzed intraoperative adjunctive strategies were not significantly associated with reduced CR-POPF in exploratory models. These findings support an individualized, risk-stratified approach to pancreatic anastomotic protection and underscore the need for larger prospective studies to define the role of specific technical adjuncts in high-risk patients.
